# An efficient power decoupling topology circuit based on a novel three-port three-switches flyback series circuit

**DOI:** 10.1371/journal.pone.0305773

**Published:** 2024-08-01

**Authors:** Wang Jinpeng, Kang Yufang, Yao Qingxue, Jeremy Gillbanks, Zhao Xin

**Affiliations:** 1 School of Information Science & Engineering, Dalian Polytechnic University, Dalian, Liaoning, China; 2 School of Electronic, Electrical and Computer Engineering, The University of Western Australia, Perth, Australia; Borgwarner, GERMANY

## Abstract

Both filter inductors, electrolytic capacitors, and radiators play a significant role in the inverter of a PV (Photovoltaic) power generation system. These three parts are the largest in an inverter, which affects the performance of the inverter. Aimed to improve the power density of a single-phase PV grid-connected inverter with a decoupling function. This paper derived the control principle that can reduce the volume of the inductor, decoupling capacitor, and the loss of the switching device to begin with the mathematical function of power processing of the filter inductor. And then, the authors deduced a boost-type power decoupled single-phase inverter topology. Based on a novel three-port three-switches flyback series circuit, this paper proposed an efficient power decoupling topology circuit for extracting the maximum power density of a single-phase grid-connected PV inverter. Finally, this article operated the simulation and experiment. Both the simulated and experimental results verified that the proposed method works well.

## 1. Introduction

Single-phase grid-connected inverters are widely attractive in distributed photovoltaic power generation, wind power generation, and other fields. In these application fields, new requirements are put forward for inverter devices achieving higher power density under high efficiency [1, 2]. The nonlinearity, randomness, and uncontrollability of wind speed and solar irradiance make inputting voltage of the inverters deviate from its given or rated value [3, 4]. The factors will lead to a significant power loss. To solve this problem, improve the adaptability to a wide range of input voltage and optimize the system reliability, flyback inverter, multi-stage inverter, and boost single-phase inverters with input voltage regulation functions have received wide attention [5, 6].

Nowadays, there are three types of inverters in PV systems, including string inverters [7], ac/AC (Alternating Current) modules [8], and centralized inverters [9]. AC module has gotten a lot of attention from the industry and researchers due to its merits: 1. an improved system efficiency; 2. enhanced modularity and flexibility; 3. a plug-n-play operation; 4. improved energy harvest; 5. low installation costs. Thanks to the advantages above, this ac module has been a trend of development for future PV systems [10].

To get the MPPT (Maximum Power Point Tracking), better the performance of the inverter, and put a specific sinusoidal current into the grid, there are two main types of decoupling circuits, including 1. the active decoupling circuits with semiconductor switches and 2. the passive decoupling ones [11–13].

In recent years, researchers have proposed several active power decoupling ways to address this problem. In the power decoupling of the output side, the system usually needs an embedding in the inverter stage of the decoupling capacitor and requires bidirectional switches [14–16]. Especially, Wang Yao [17, 18] designed a differential buck converter. In this converter, the filter capacitor connects to the output terminal and the bus with the negative dc/DC (Direct Current) for storing pulsating energy [19, 20].

An input-side power decoupling has some capacitors placed on the PV side, and these capacitors have two types, including parallel and series methods. Articles [21–23] give the concept of the series power decoupling in different ways. The parallel power decoupling using the active filter idea is introduced in [24–26]. Shimizu etc. [27] have researched a type of flyback AC-link inverter with a double-stage. Articles [28, 29] introduced a specific power decoupling method with a six-switch inverter to recycle some energy from leakage inductance.

Paper [30] has also proposed a modified version of the power decoupling way using a five-switches to solve it. Furthermore, a research report [31, 32] has suggested a combination algorithm of flyback and boost transformers based on a technology of capacitive idling. There are two processing steps in these microinverters mentioned above: power delivery through a power decoupling technology and the energy capture of the PV using MPPT. Shinjo [33] presented an efficient push-pull forward inverter using power decoupling capability.

Compared with parallel and series methods, power decoupling technology has gotten extensive attention because it only requires small passive components (i.g., small thin film capacitors) to deal with pulsating power without electrolytic capacitors or other power circuits [34, 35]. Consequently, scientists widely used this single-stage inverter for PV generation owing to its simple structure and low cost. However, the single-stage inverter has its shortcoming: a double power frequency pulse electromotive force will probably cause large fluctuations both in the voltage and the current at the PV side [36], resulting in difficulties during the design of the MPPT controllers. In addition, the existing passive decoupling scheme using electrolytic capacitors leads to the shortening of the service life of the inverter.

Furthermore, the inductance coil of the transformer is large and heavy, and the power density of the inverter is low in the flyback grid-connected inverter topology [37]. Although the passive inverter has some advantages of small size [38], low cost [39], simple structure [40], and high efficiency mentioned above, its power density is still low due to the electrolytic capacitor in the single-phase inverter.

This paper proposed an efficient power decoupling topology circuit for extracting the maximum power density of a single-phase grid-connected PV inverter based on a novel three-port three-switches flyback series circuit to address the problems mentioned above. The proposed method can deal with power differences between the output and input using some small thin-film capacitors joined with the modified three-port three-switch flyback converter. It practices power decoupling only through three diodes and three buttons. To our knowledge, the presented way possesses the least number of switching controls among microinverters and the inverters proposed before.

The structure of the paper is as follows: section II gives the traditional single-phase inverter topology and its grid-connected flyback inverter; section III proposes the topology of the three-port three-switches flyback series circuit; part IV shows operation modes and control strategy; section V illustrates the simulation and experiment performed in this paper as well as their analysis; finally, section VI concludes the whole research.

## 2. Traditional single-phase inverter topology and its grid-connected flyback inverter

During a definite switching period in the topology of this paper, AC power refers to the charging or discharging of an inductance; DC power refers to the power transmitted directly from the power source to the load end in this period. Consequently, the sum of AC power and DC power is the output power in this period.

**Fig 1:**
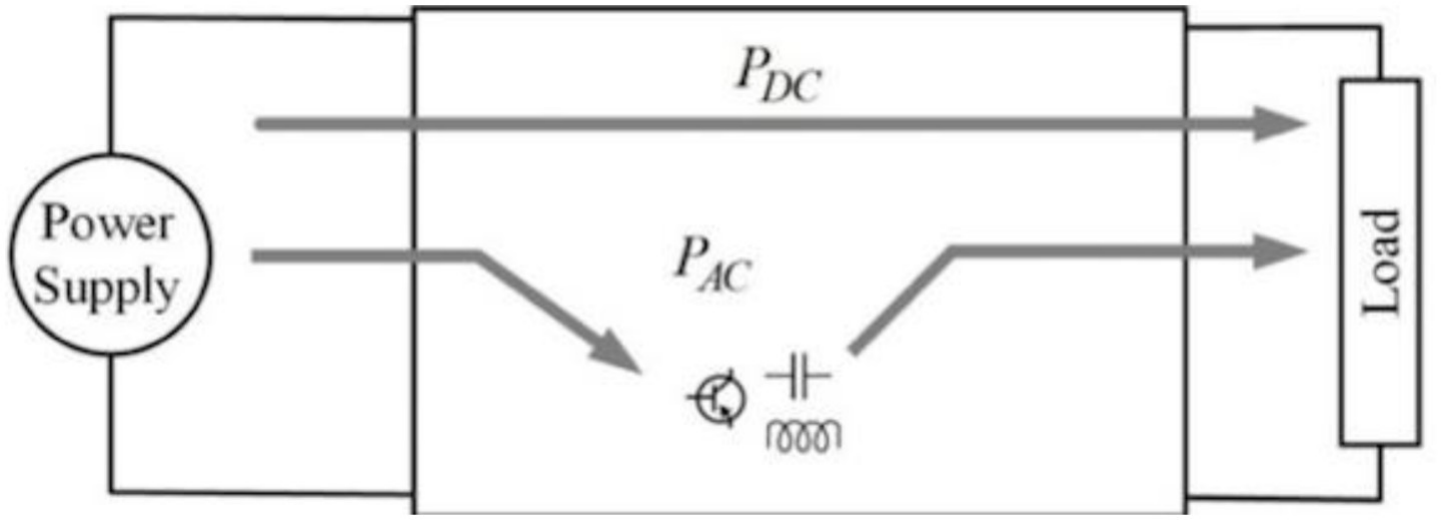
A power flow of the topology.

As shown in Fig 1 below, DC power ***P***_*DC*_ denotes the power transferred directly from the power source to the load without the volume or loss of power converter components; AC power ***P***_*AC*_ refers to the one transmitted to the load circuit when reactive devices finished processing or converting.

### 2.1. Traditional grid-connected flyback type inverter

**Fig 2:**
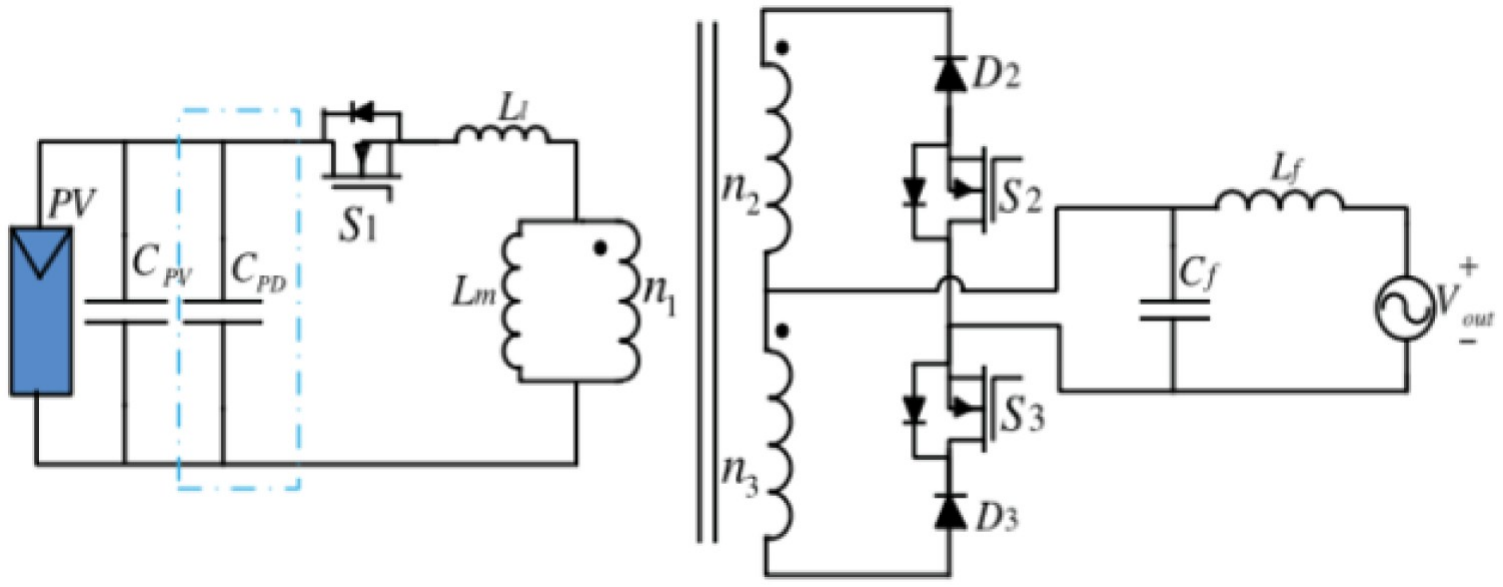
A traditional grid-connected flyback inverter.

Fig 2 gives one traditional grid-connected flyback type inverter [41]. The inputting power ***P***_***PV***_ is one constant value decided via the MPPT method. The output power ***P***_***out***_ **(*t*)** is a variable that varies with time and consists of the AC and DC parts. Supposing this inverter as lossless, the injected current and the grid voltage to be sinusoidal in-phase waveforms, the DC aspect of the outputting power is the inputting power, and the AC aspect fluctuates in a double-line frequency.

Consequently, the real-time inputting power and instantaneous outputting power are as expressions (5,6) below:

$$P_{PV}=V_{PV}∙I_{PV}$$ (1)

$$P_{out}\left(t\right)=V_{m}∙I_{m}{sin}^{2}\omega t=P_{PV}∙\left(1-cos2\omega t\right)$$ (2)

The system processes the fluctuation aspect of the outputting power passing through one buffer to achieve a constant DC on the inputting side. This instantaneous power in the buffer equals Eq (7) below:

$${P_{PD}\left(t\right)=P}_{PV}∙cos2\omega t$$ (3)

Different from the meanings of ***P***_*DC*_ in Fig 1 (where it refers to the power transferred directly from the power source to the load without the need for a power converter or loss of power). The parameter ***P***_PD_ in Eq (3) represents the power delivered by decoupling circuits, while the abbreviation PD stands for Power Decoupling.

The system puts a big electrolytic capacitor *C*_PD_ across the PV to control the voltage fluctuation in one passive power decoupling circuit. Nevertheless, the electrolytic capacitor is commonly sensitive to the environment’s temperature and could go down the overall reliability of the inverter.

**Fig 3:**
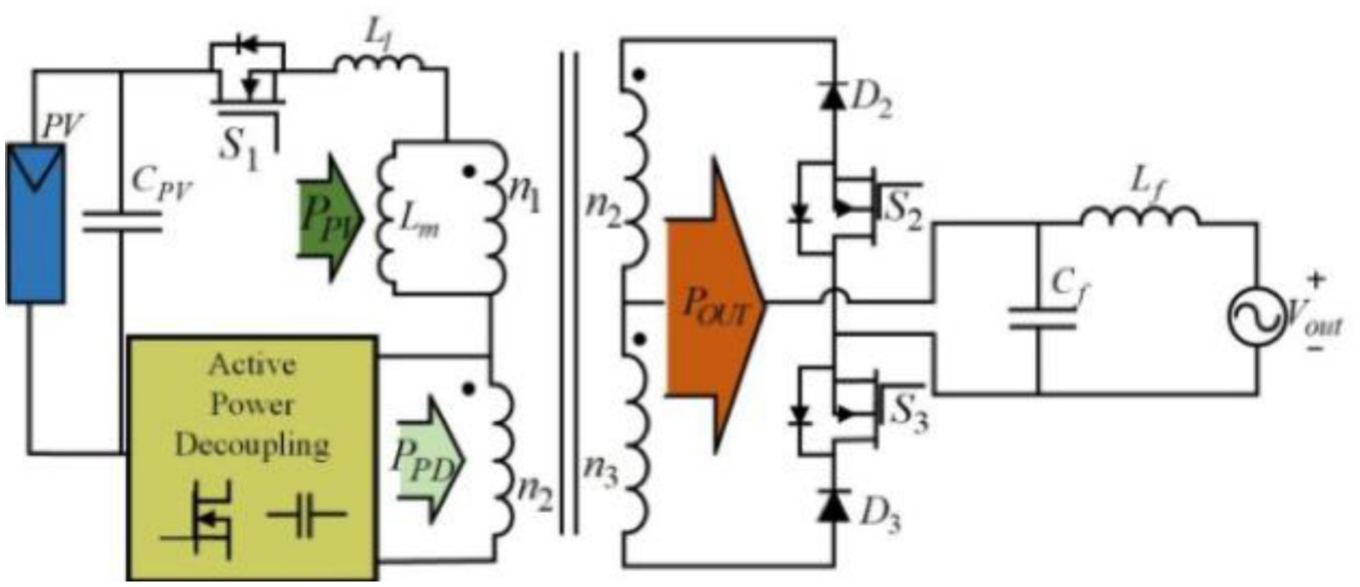
A traditional flyback inverter fitted with a power decoupling circuit.

Fig 3 below illustrates a traditional flyback inverter fitted with a power decoupling circuit. The technology of active power decoupling is the other to compensate for power differences using active switches and long-lifetime thin film capacitors. If the inputting power is more than the outputting one, then the system will transport the extra energy to the decoupling capacitor from the PV. While the inputting power is less than the demanded grid power, this energy of the decoupling capacitor transports to the outputting end. In the three-port power decoupling circuit, by using a port for the power decoupling but applying the rest two ports for obtaining the inputting power and conveying this power into the outputting port.

There are two operating states in the inverter based on this power difference among the instantaneous outputting and inputting powers, including State I and II. On the one hand, in State I, the inputting power is less (at least no more) than the outputting power, and that decoupling capacitor is discharging. Then again, during State II, the inputting power is more (at least no less) than the outputting power, and the decoupling capacitor charges via absorbing the energy. Because this decoupling circuit only deals with the pulsating power, so average power of the decoupling topology should be zero.

## 3. Proposed topology of the three-ports three-switches

**Fig 4:**
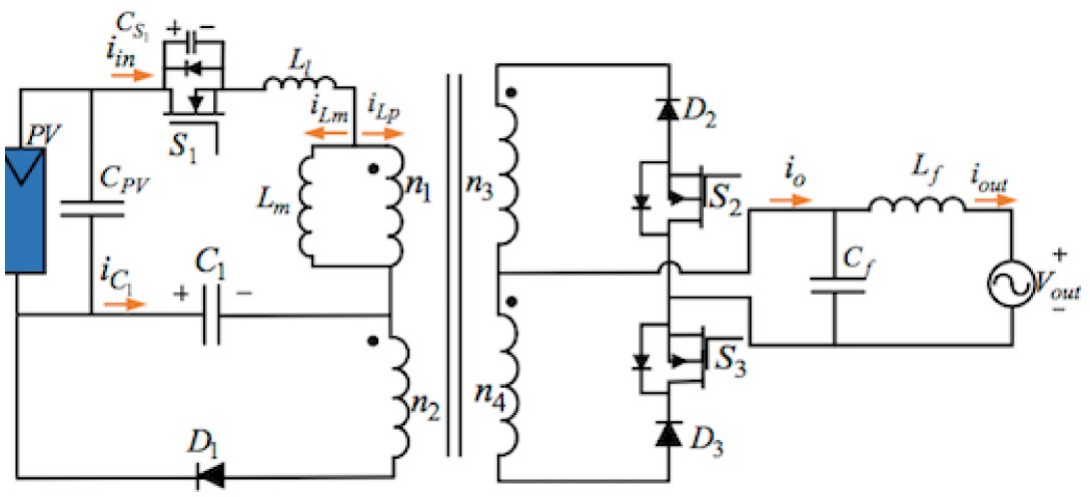
Model of the proposed topology of the three-port three-switches flyback series circuit.

Fig 4 below illustrates the proposed topology of the three-port three-switches flyback series circuit. The presented AC module is an improved single-phase flyback type inverter, which is a modified circuit deduced from the traditional flyback inverter by integrating an active power decoupling circuit and another transformer winding. The proposed inverter with three switches can get maximum power from the PV based on the MPPT method, input the sinusoidal current into the grid, and compensate for the differences between the outputting and inputting powers through one little thin-film capacitor. Consequently, just operating switches S_1_, S_2_, and S_3_ can readily achieve those functions.

This power decoupling circuit includes a decoupling capacitor C_1_ and a diode D_1_. There is no individual switch to deal with the pulsating power in the proposed decoupling topology. Nevertheless, the system uses a flyback main switch S_1_ to keep the pulsating energy and the PV energy in that transformer in storage. Placing a buffer capacitor across S_1_ can decrease the electromagnetic noise and supply some soft-switching conditions. Applying two secondary-side diodes D_2_, and D_3_, as well as two switches S_2_, and S_3_, connected in series, can transfer the power to the grid from the PV using a proper transformer winding. The presented inverter topology filters some switching frequencies using an LPF (Low-Pass Filter) and transfers a low THD (Total Harmonic Distortion) current into the grid.

The inverter proposed in this article has three merits:

(1). it is a plain architecture with just one capacitor and only one diode equipped with the secondary winding of the transformer for implementing the function of the power decoupling;
(2). it can realize the MPPT, operate the power decoupling using only three switches, and put the sinusoidal current into the grid;
(3). the circuit performs in a DCM (Discontinuous Conduction Mode), which obtains merits from the soft-switching technology and just one easy controlling method.

## 4. Operation modes and control strategy

### 4.1. Operation modes

There are five modes of inverter operation within every switching cycle, where nab symbolizes the turn-ratios of the transformer: n_a_/n_b_ (a, b = 1, 2, 3, 4).

**Fig 5:**
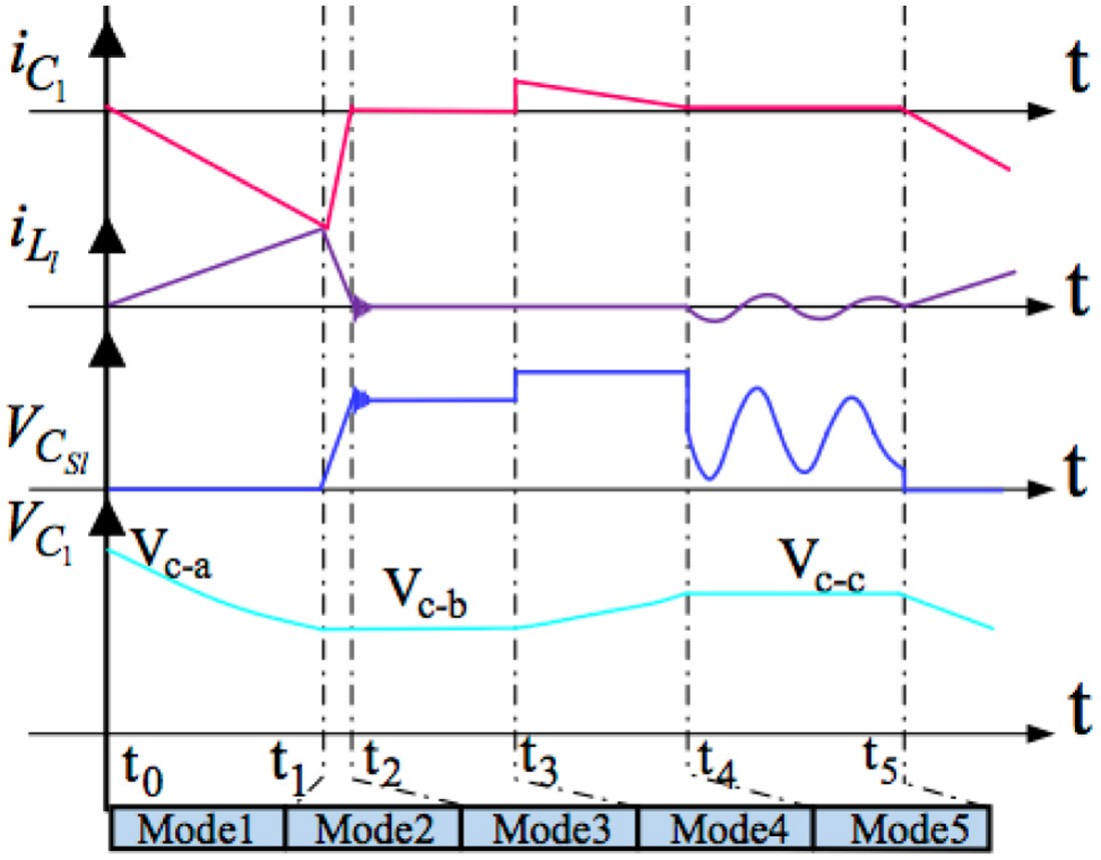
The vital waveforms of the inverter in one switching cycle.

Fig 5 below shows the vital waveforms of the inverter in one switching cycle. Because the switching frequency of the inverter is much bigger than the power system frequency, the referencing current and the grid voltage are nearly stable within one switching cycle. This paper supposed that all switches are in the state of off before the first sub-interval; in addition, the voltage of the decoupling capacitor V_C1_ equals V_c-a_; furthermore, the authors also supposed the voltages of the grid are at the upper half period.

**Fig 6:**
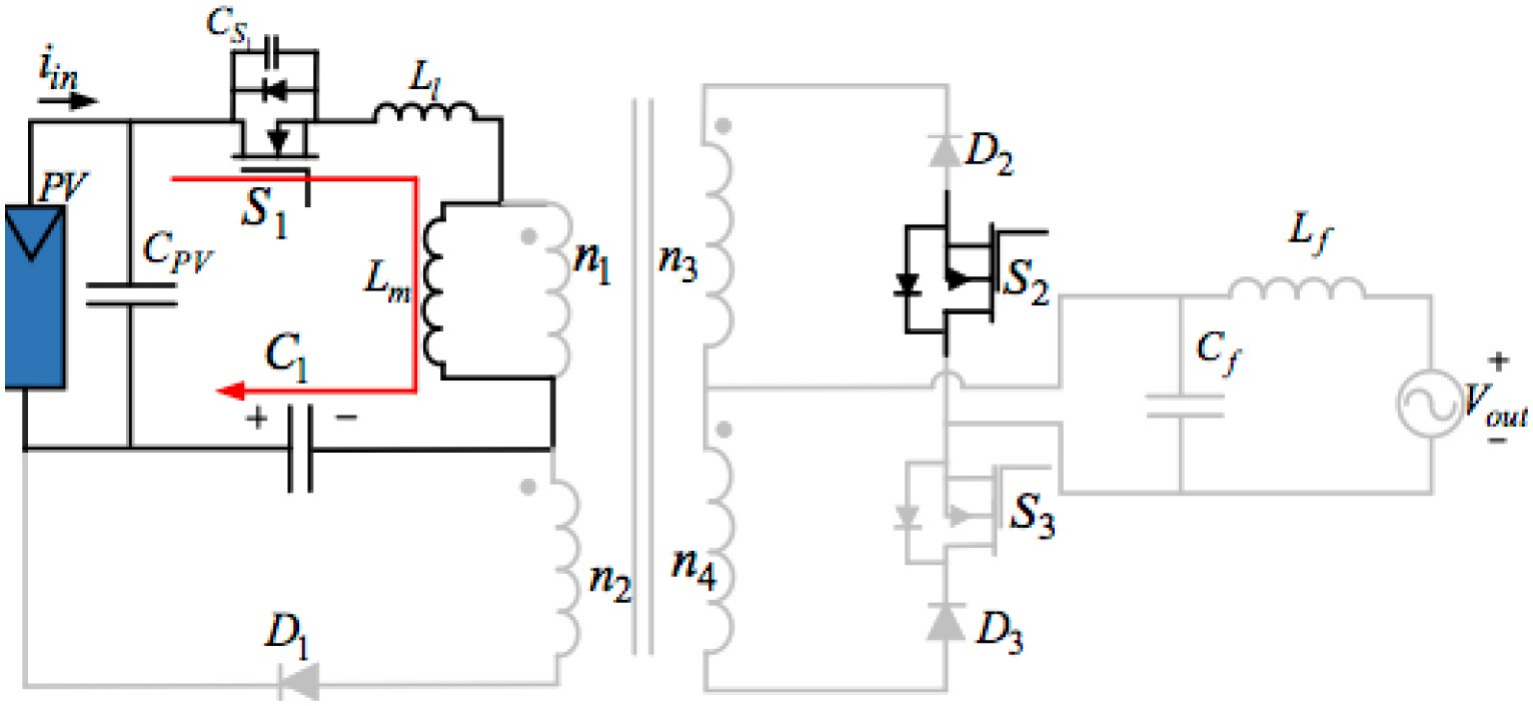
Equivalent circuit of mode 1.

**Fig 7:**
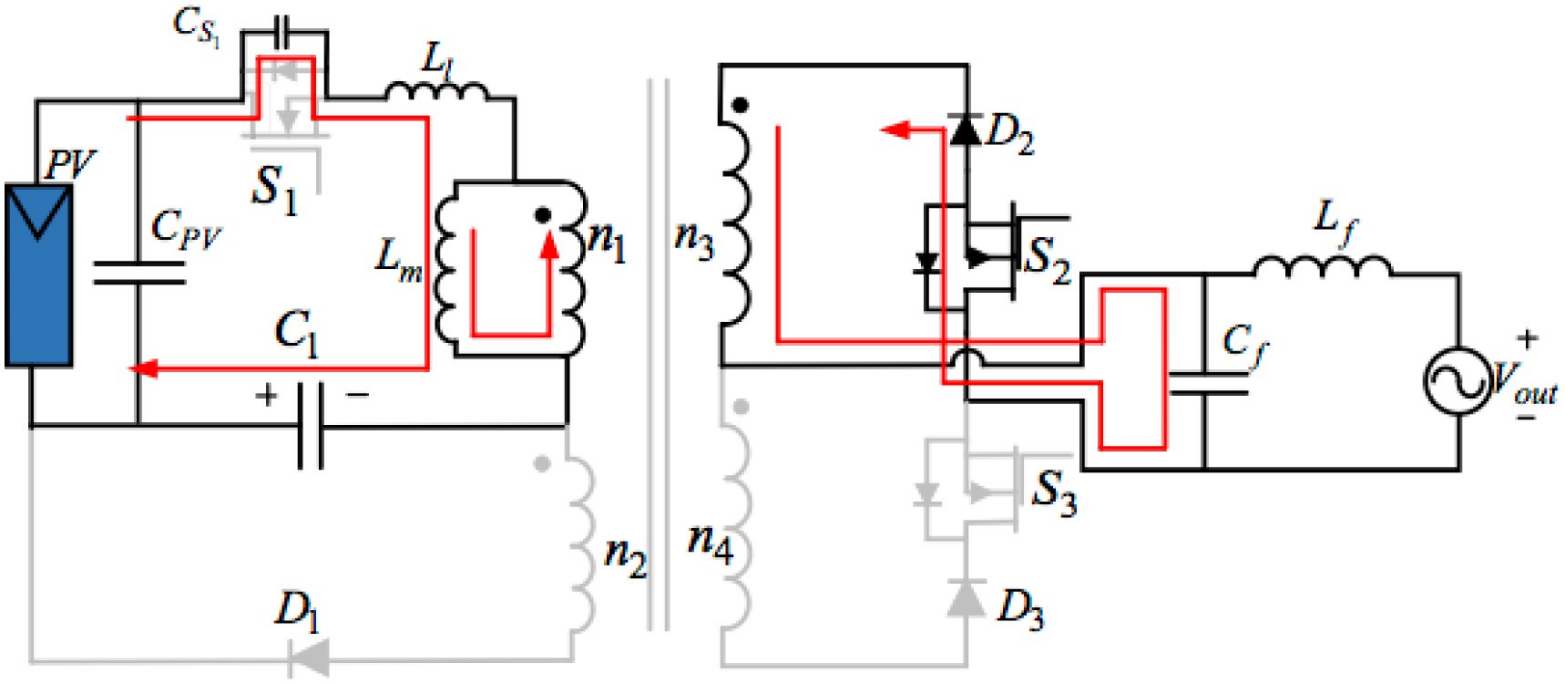
Equivalent circuit of mode 2.

**Fig 8:**
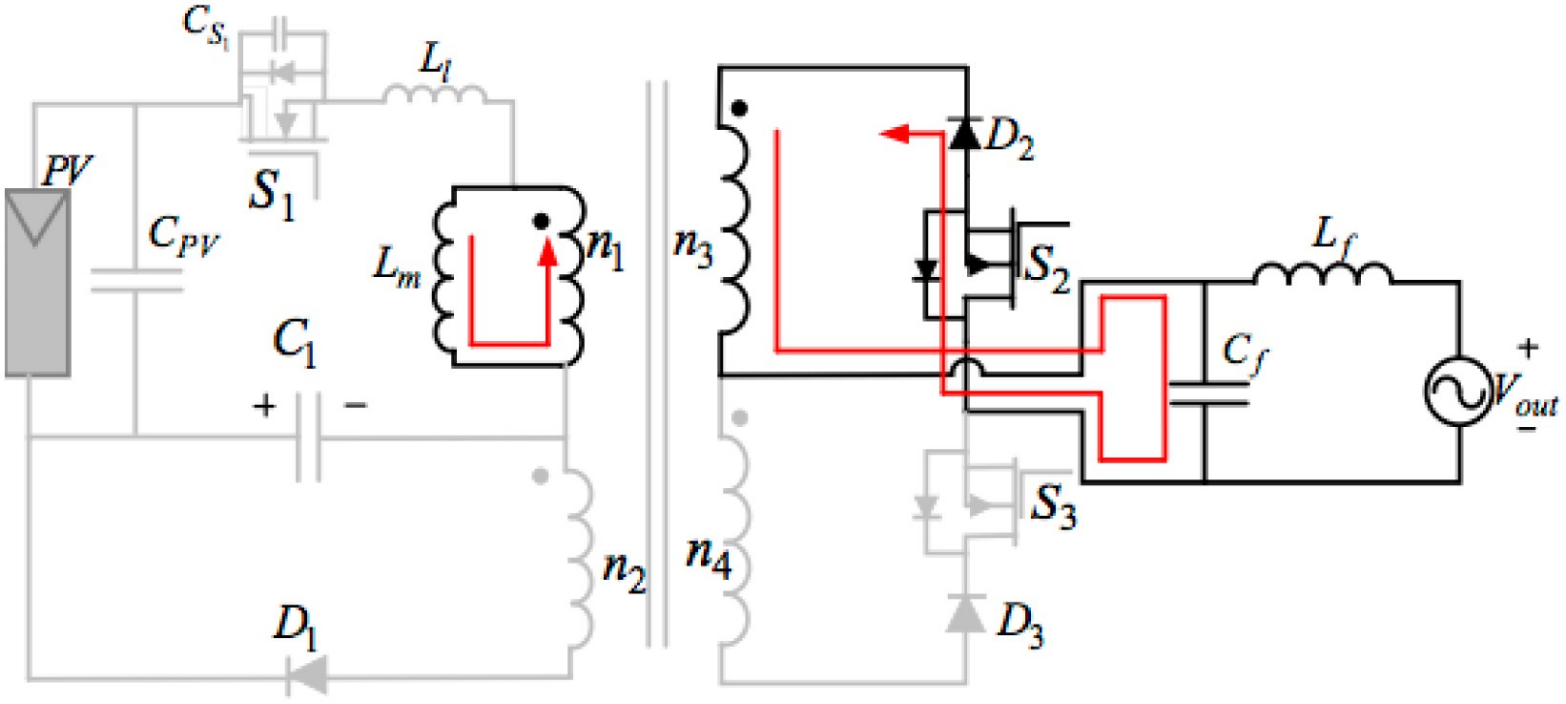
Equivalent circuit of mode 3.

**Fig 9:**
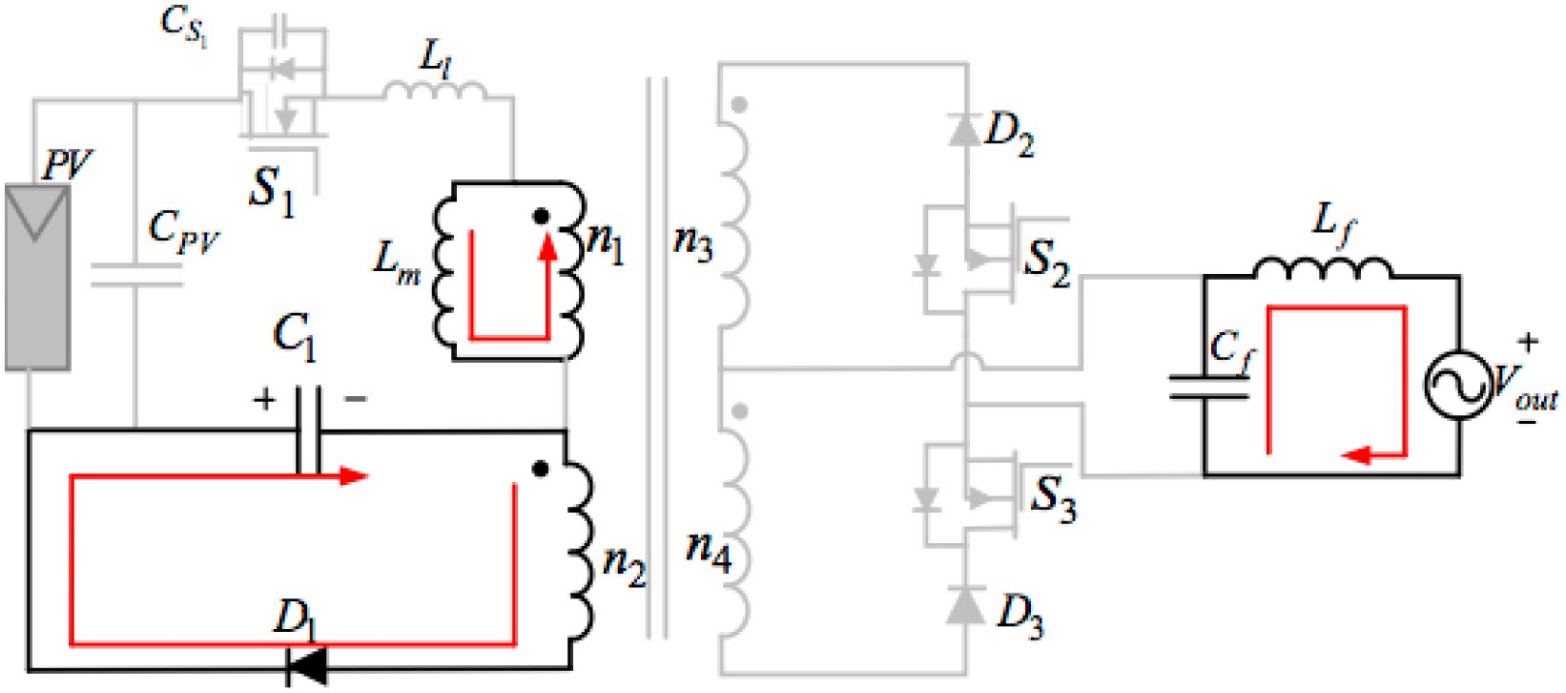
Equivalent circuit of mode 4.

**Fig 10:**
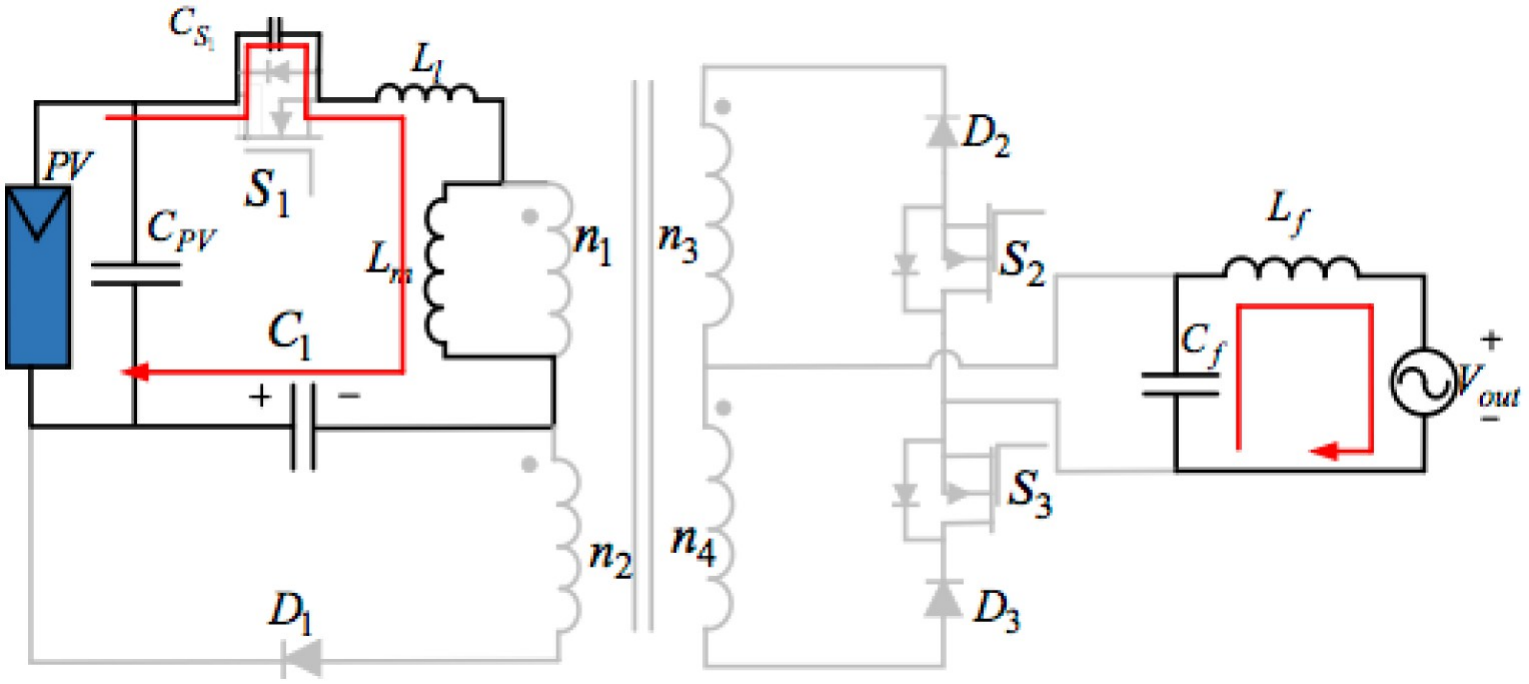
Equivalent circuit of mode 5.

Accordingly, the following parts describe all five operation modes of the proposed inverter in Figs 6–10:

- MODE 1 (PERIOD BEING t_0_< t < t_1_)
  Fig 6 below shows the equivalent circuit of operation mode 1. The magnetizing inductance of the transformer L_m_ and the buffer capacitor C_S1_ are both in the state of resonance before mode 1. At time t_0_, turning off switches S_3_ and turning on S_1_ and S_2_, when that voltage bypassed switch S_1_, meets the minimum in its resonance.
  Consequently, the circuit will turn on switch S_1_ close to the condition of the ZVS (Zero Voltage Switching). Switch S_2_ conducts no current despite turns on it because this series diode D_2_ is reversed-biased. Therefore, the circuit turns on switch S_2_ under the condition of the ZCS (Zero Current Switch). Applying the sum of the decoupling capacitor and the PV cross magnetizing inductances and the transformer leak in this mode is vital. Since the proposed circuit works in the DCM(Discontinuous-Conduction Mode), the current in the transformer linearly increases from the point of zero can be formulated as below:
$$i_{Lm}\left(t\right)=\frac{V_{PV}+V_{C_{1}}}{L_{m}+L_{l}}(t-t_{0})$$ (4)
  Mode 1 will not stop until turning off switch S_1_ at the time of t_1_. The system delivers some portion of the energy of the decoupling capacitor into the magnetizing inductance in the transformer. Consequently, this voltage will go down to V_c-b_ from V_c-a_ when mode1 ends. Thus, we can describe the voltage of the decoupling capacitor at the end of this mode as Eq (9) below:
$$V_{c-b}=\sqrt{V_{c-a}^{2}+\frac{2d_{1}V_{PV}I_{PV}}{C_{1}f}-\frac{L_{m}}{C_{1}}I_{peak11}^{2}}$$ (5)
  In the expression above, $P_{PV}=\frac{1}{2}i_{peak11}d_{1}V_{PV}$ symbolizes the inputting power; $i_{peak11}=\frac{V_{PV}+V_{C_{1}}}{\left(L_{m}+L_{l}\right)f}d_{1}$ means the maximum value of the magnetizing inductance current in the specific transformer; and $d_{1}=\sqrt{\frac{2P_{PV}\left(L_{m}+L_{l}\right)f}{V_{PV}(V_{PV}+V_{C_{1}})}}$ denotes the duty cycles of switch S_1_.
- MODE 2 (PERIOD BEING t_1_< t < t_2_)
  Fig 7 below gives the equivalent circuit of operation mode 2.
  Because the system places capacitor C_S1_ across S_1_, switch S_1_’s voltage smoothly increases and will be turned off in the condition of the AVS. Compared with a state of the art that limits the voltage change by putting a large electrolytic capacitor CPD across a PV in the passive power decoupling, which could decline the reliability of the inverter due to the temperature sensitivity, the proposed model can ignore the smooth little magnitude increase of switch S1’s voltage through using an active power decoupling. This increasing voltage starts from the value of zero and lastly ends with a value of V_SS_:
$$V_{SS}=V_{C_{1}}+V_{PV}+n_{13}V_{out}$$ (6)
  This current of the leakage inductance in the transformer slowly goes down during mode2. and will be zero when mode 2. ends. The authors consider the magnetizing inductance constant because the magnetizing inductance is much bigger than the leakage one, and the lasting time of mode 2 is so short. Formulas (7,8) below respectively symbolize the voltage of switch S_1_ and the leakage inductance current in the transformer:
$$i_{L_{l}}\left(t\right)=i_{peak11}cos\left(\omega_{0}\left(t-t_{1}\right)\right)+\frac{V_{SS}}{Z_{1}}sin\left(\omega_{0}\left(t-t_{1}\right)\right)$$ (7)
$$V_{C_{S1}}=V_{SS}\left[1-cos\left(\omega_{0}\left(t-t_{1}\right)\right)\right]+\left(i_{peak11}Z_{1}\right)sin\left(\omega_{0}\left(t-t_{1}\right)\right)$$ (8)
  Where, $\omega_{0}=\frac{1}{L_{l}C_{S1}}$ *and* $Z_{1}=\sqrt{\frac{L_{l}}{C_{S1}}}$.
- MODE 3 (PERIOD BEING t_2_< t < t_3_)
  Fig 8 below illustrates the equivalent circuit of operation mode 3. The voltage of the decoupling capacitor is unchanged in this mode.
  Fig 8 shows that turning S_2_ off at the time of t3 can terminate mode 3. During mode3, switch S_2_ remains on, and by operating diode D2 and switching S_2_ transfers some parts of the energy stored in the proposed flyback transformer into the grid. A peak value of the outputting current in the transformer at the start of mode 3 is:
$$i_{peak21}=n_{13}\frac{V_{PV}+V_{C_{1}}}{\left(L_{m}+L_{l}\right)f}d_{1}$$ (9)
  The outputting current of the magnetizing inductance at the end of mode3. could be related to its initial value from starting this mode. A minimum value of the outputting current in the transformer at the start of mode 3 is:
$$i_{peak22}^{2}=i_{peak21}^{2}-\frac{2n_{13}^{2}V_{m}I_{m}{sin}^{2}\omega t}{fL_{m}}$$ (10)
- MODE 4 (PERIOD BEING t_3_< t < t_4_)
  Fig 9 below denotes the equivalent circuit of operation mode 4.
  At the time of t_3_, the circuit turned S_2_ off and charged the decoupling capacitor using the rest energy in the flyback transformer via diode D_1_ and the second winding of the transformer. The current of the magnetizing inductance from the starting of mode 4 equals as expression (11) below:
$$i_{peak12}=i_{peak22}/n_{13}$$ (11)
  When mode 4. ends, the voltage of the decoupling capacitor could increase to *V*_*c-c*_:
$$V_{c-c}=V_{c-b}+\frac{L_{m}L_{peak12}^{2}}{2n_{12}C_{1}V_{C_{1}}}$$ (12)
  In addition, formula (13) below represents the voltage of switch S_1_:
$$V_{dd}=V_{PV}+(1+n_{12})V_{C_{1}}$$ (13)
- MODE 5 (PERIOD BEING t_4_< t < t_5_)
  Fig 10 below denotes the equivalent circuit of operation mode 5. The system goes to this mode when the energy in the transformer completely discharges into the decoupling capacitor at time t_4_.

At this moment, resonance occurs among the transformer magnetizing and the buffer capacitor, as well as leakage inductance. Expressions (14,15) below, write the buffer voltage and the inductance current:

$$V_{C_{S1}=}V_{pp}+n_{12}V_{C_{1}}cos(\omega_{0}^{'}(t-t_{4}))$$ (14)

$$i_{L_{l}}\left(t\right)=-(n_{12}V_{C_{1}}/Z_{1}^{'})sin(\omega_{0}^{'}(t-t_{4}))$$ (15)

In the equations above, $Z_{1}^{'}=\sqrt{\left(L_{m}+L_{l}\right)/C_{S1}}$ is the characteristic impedance in mode 5, $\omega_{0}^{'}=1/\sqrt{\left(L_{m}+L_{l}\right)/C_{S1}}$ denotes the angular frequency of the grid voltage, and inductance ${V_{PP}=V}_{PV}+V_{C_{1}}$ represents the steady-state voltage of switch S_1_ at this mode.

### 4.2. Control strategy

**Fig 11:**
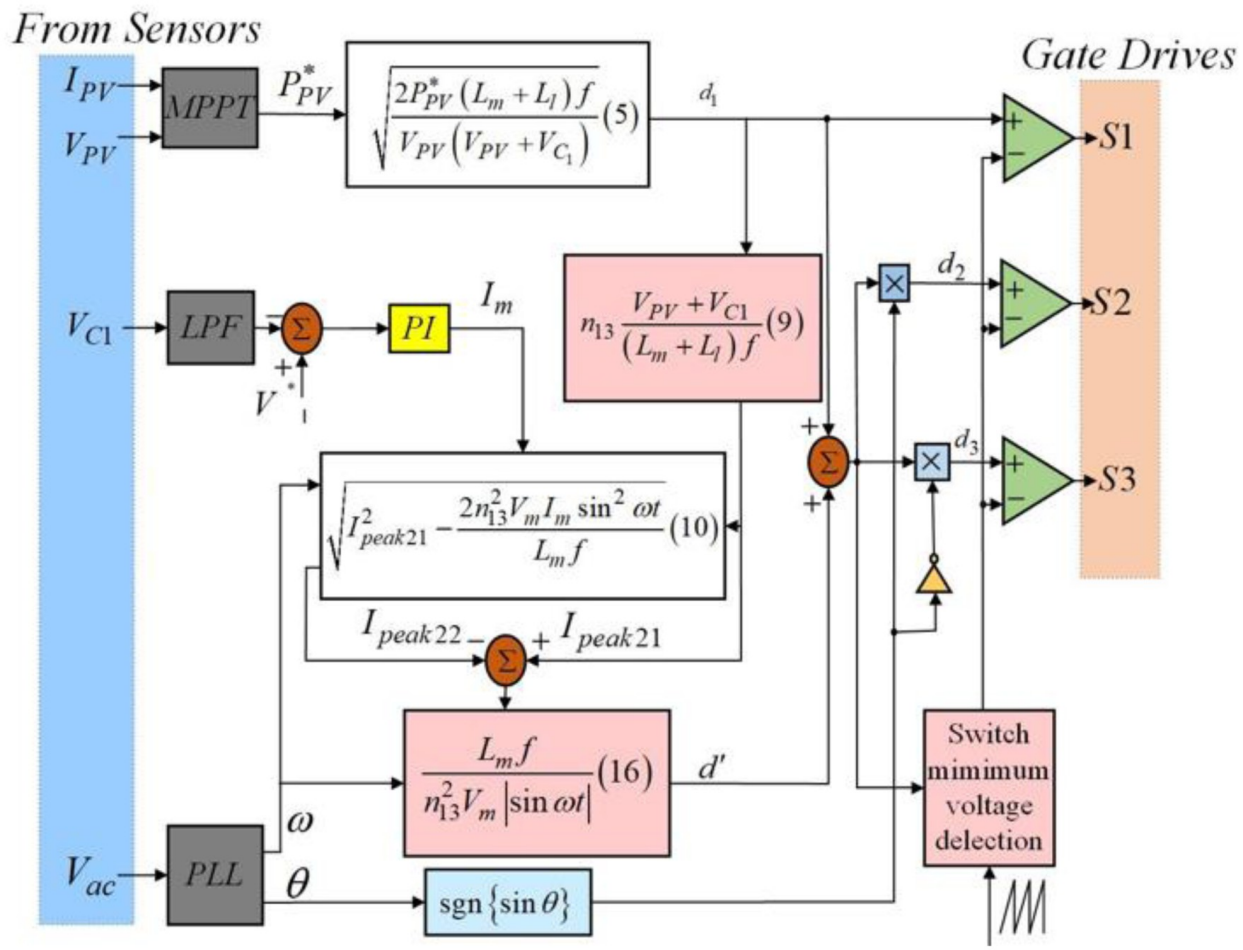
Block diagram of the controlling strategy of the designed inverter.

Fig 11 illustrates a block diagram of the presented flyback inverter. There are five functional modules: a PLL(Phase-Locked Loop), voltage sensors, half-cycle detection, one outputting current controller, and an MPPT controller. As shown in this figure, the proposed circuit can measure some parameters, including the grid voltage *V*_*ac*_, the decoupling capacitor $V_{C_{1}}$, the PV current *I*_PV_, and the voltage of PV *V*_PV_. The controlling strategy designed here ensures the system can abstract the MPP in the PV and transfer this maximum power into the grid in good quality.

An MPPT controller can abstract the maximum power stored in the PV panel based on an incremental conductance algorithm. The proposed inverter works under the condition of the DCM. Turning switch S1 on can store the energy in the flyback transformer through the decoupling capacitor and the PV. It is better to determine the duty cycle d_1_ of switch S1 based on the MPPT method to satisfy the definition (i.e., $d_{1}=\sqrt{\frac{2P_{PV}\left(L_{m}+L_{l}\right)f}{V_{PV}(V_{PV}+V_{C_{1}})}}$).

In this meaning above, *L*_*m*_ stands for Transformer magnetizing inductance, and *L*_*l*_ represents Transformer leakage inductance. *L*_*m*_ and *L*_*l*_ are inversely proportional to the Duty cycle of switch S1 (i.e., *d*_1_) but proportional to the Maximum current of transformer output winding during mode 3 (i.e., *i*_peak21_), according to Fig 13, expression (5) and equation (9).

Furthermore, as far as *L*_*m*_ is concerned, it is inversely proportional to the Minimum current of transformer output winding during mode 3 (i.e., *i*_peak22_) but proportional to Subtraction of d2 (d3) and d1 (i.e., *d*’ = *d*_2_ − *d*_1_) based on Fig 13, formula(10) and expression (16).

Employing a block of PLL can determine the phase angle of the voltage because the grid voltage should be in phase with the outputting current. Using this module also can recognize the voltage negative and positive half cycle. The inverter will control S_2_ and quench S_3_ when the grid voltage is in the positive half cycle. Conversely, it tends to turn switch S_2_ off and control switch S_3_ when the voltage locates at its negative half cycle. The duty cycle of switches (S_2_ or S_3_) equals a sum of d_1_ and d’ deduced by Eq (9):

$$d^{'}=\frac{L_{m}f}{n_{13}^{2}V_{m}\left|sin\omega t\right|}\left(i_{peak21}-i_{peak22}\right)$$ (16)

## 5. Results and analysis

### 5.1. Simulation results and analysis

This article built a simulating topology circuit with 750 Watts in MATLAB to verify the proposed method’s performance. Table 1 below lists the main simulation parameters of the proposed topology.

**Table 1:** Simulation factors of the proposed topology.

Parameters	Units	Value
Grid voltage	V	312
Grid current	A	5
Rated power	W	760
Filter inductance	*μH*	0.9
Flyback inductance	*μH*	210
Decoupling capacitor	*μF*	165
Grid frequency	Hz	50
Switching frequency	kHz	15

**Fig 12:**
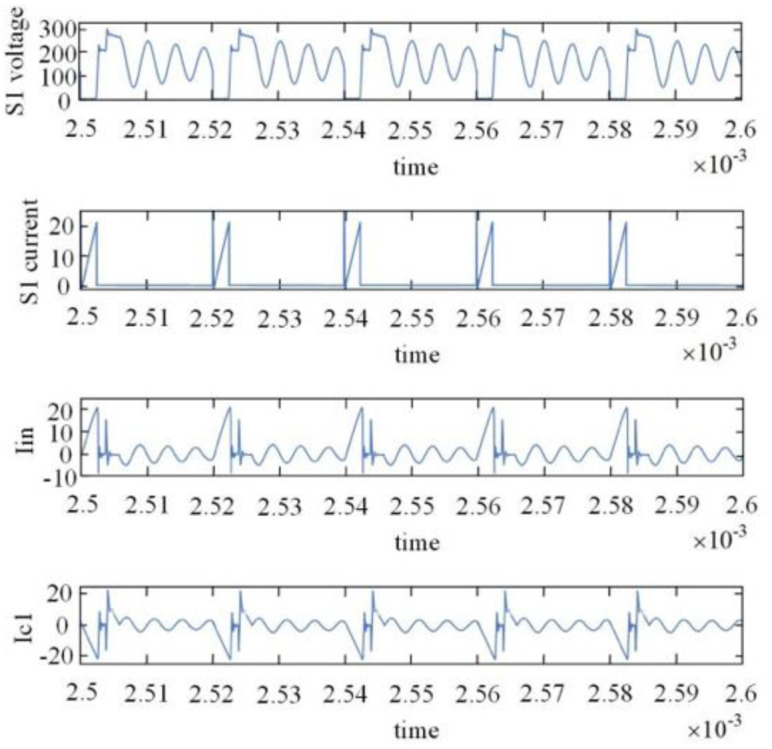
Voltage across switch S_1_, current of switch S_1_, inverter input current, and current of a decoupling capacitor.

**Fig 13:**
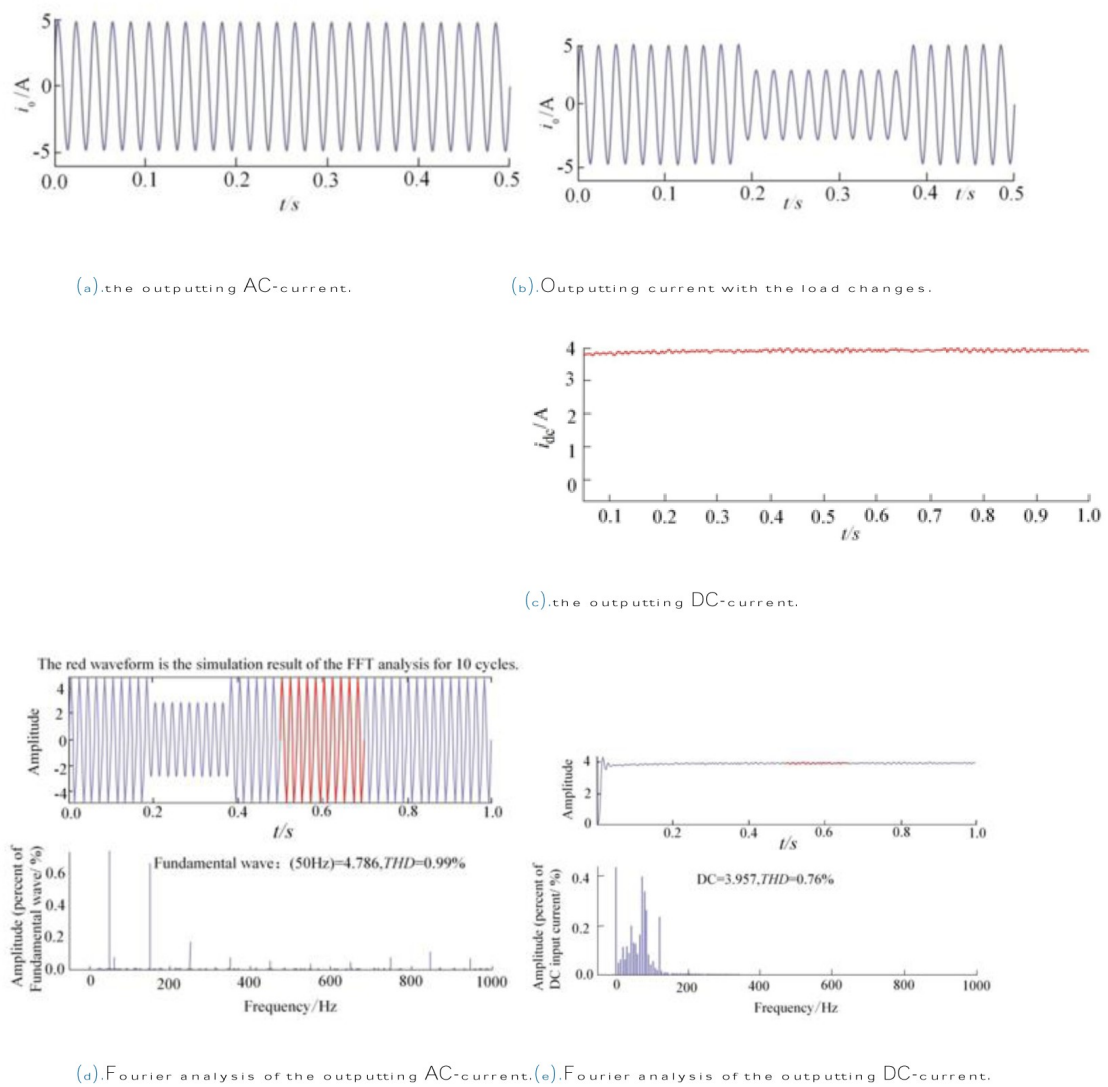
Results of simulation for the robustness. (a). the outputting AC-current. (b). Outputting current with the load changes. (c). the outputting DC-current. (d). Fourier analysis of the outputting AC-current. (e). Fourier analysis of the outputting DC-current.

**Fig 14:**
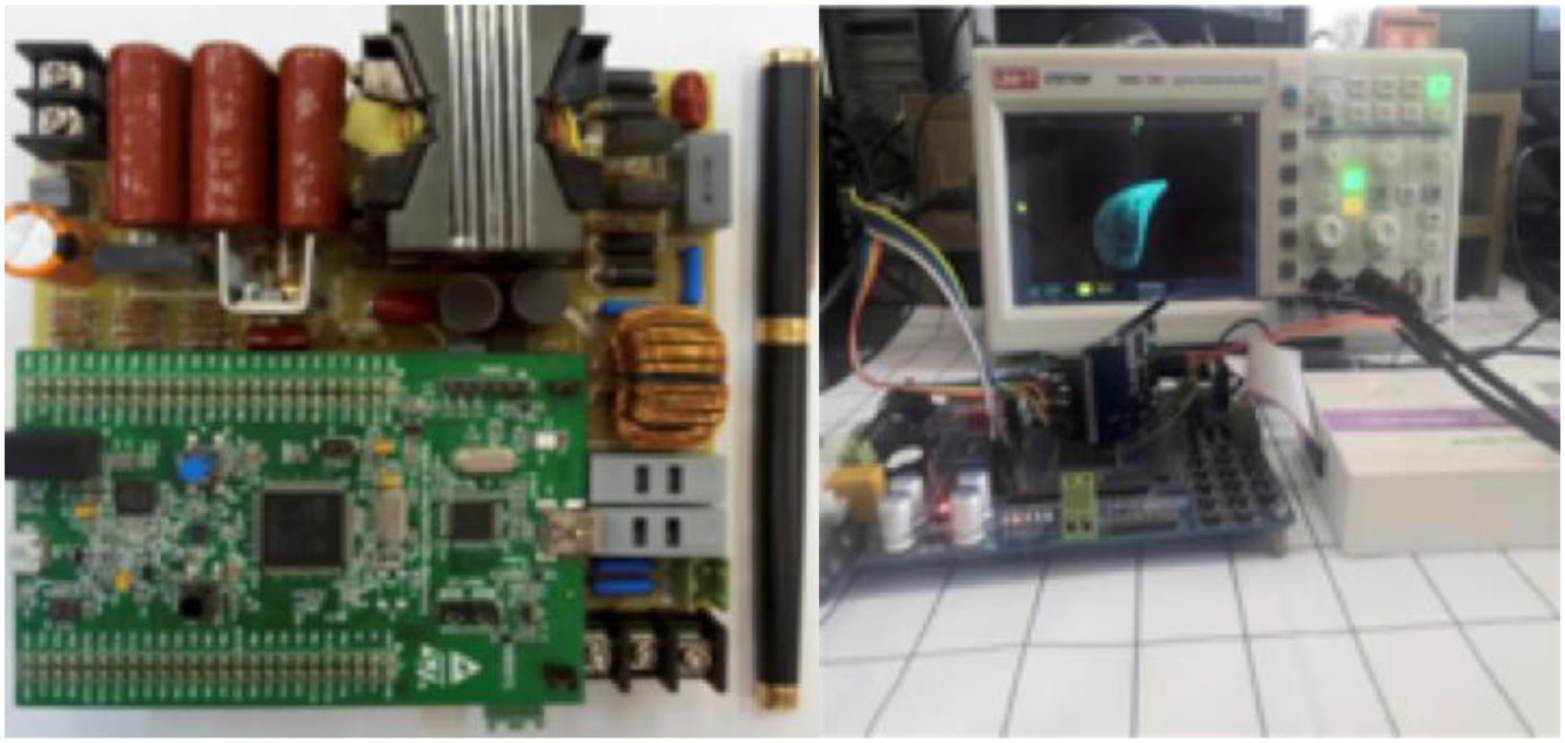
Experimental prototype.

Figs 12 and 13 show the simulation results. Fig 14 below illustrates some different current and voltage waveforms according to the genuine values of parameters. Via turning off switch S1, the voltage of the S1 slowly increases owing to the buffer capacitor. Accordingly, the circuit will turn switch S1 OFF in the condition of the ZVS.

In addition, Fig 13 demonstrates the other simulation results from a different perspective:

### 5.2. Experimental results and analysis

To test the performance of the presented inverter, we implemented a paradigm inverter with the 100 watts shown in Fig 14. The experiment selected an STM32F407VGT6 equipped with a 32-bit MCU of an ARM Cortex-M4 as the core in implementing this digital controller. In addition, the design adopted a PV simulator as an inputting power source.

Table 2 listed the critical parameters and corresponding values used in the circuit, and Table 3 specified those switches and diodes.

**Table 2:** Simulation factors of the proposed topology.

Design Parameters	Value
The voltage of the AC grid *V*_AC_	220V
The frequency of the AC grid voltage *f*_ac_	50Hz
The inputting voltage of the DC power supply *V*_PV_	60V
The switching frequency *f*	50Hz
The nominal outputting power	100W
The turn-ratios of the transformer (*n*_1_: *n*_2_: *n*_3_: *n*_4_)	1:1:4:4
The turn-ratios of the transformer L_*m*_	50*μH*
The inductance of the filter L_*f*_	1*mH*
The buffer capacitor of the switch *S*_1_ *C*_*S*1_	4.7*nF*
The capacitor of the filter *C*_*f*_	15*μF*
The capacitor placed across PV *C*_*PV*_	35*μF*
The capacitor the power decoupling *C*_1_	80*μF*

**Table 3:** Parameters of the switches and diodes.

Diodes or switches	Item number	parameters
*D*_1_	STTH1602C	*t*_rr_ = 21*ns*, *I*_*f*_ = 2 × 10A, *V*_*D*_ = 200V
*S*_1_	IRFP4229PbF	*V*_DS_ = 300V, *R*_DS_ = 38mΩ
*D*_2_&*D*_3_	UF4008	*t*_rr_ = 75*ns*, *I*_*f*_ = 1A, *V*_*D*_ = 1000V
*S*_2_&*S*_3_	STU7NB100	*V*_DS_ = 1000V, *R*_DS_ = 1.2Ω

**Fig 15:**
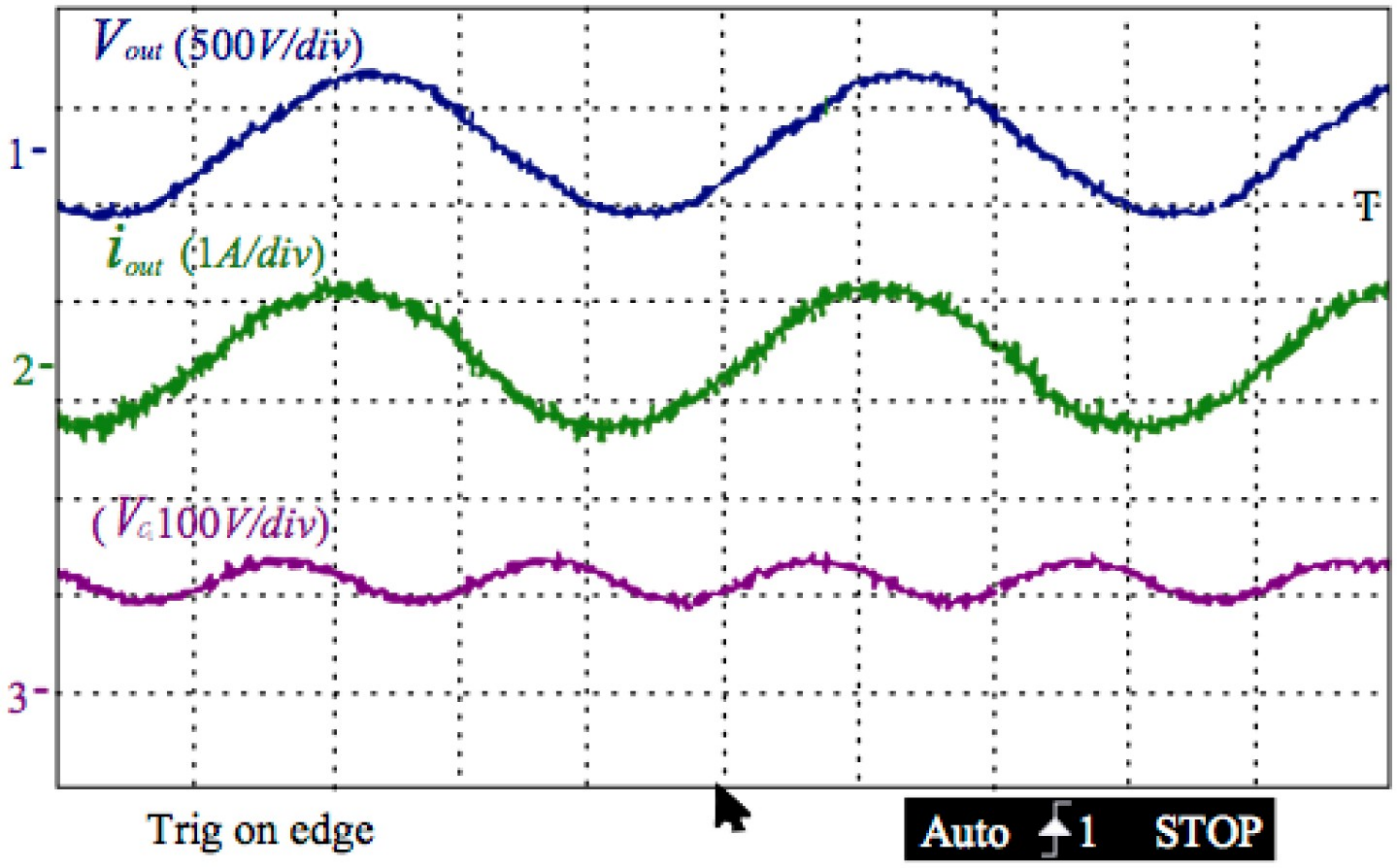
Outputting current, the grid voltage, and the voltage of the decoupling capacitor.

Fig 15 below illustrates the experimenting production of the outputting current, the grid voltage, and the voltage of the decoupling capacitor. A power analyzer of C.A.8335 surveyed the outputting current’s THD is 3.5%. As shown in the figure, the output current is one sinusoidal signal with the same phase as the grid voltage. As shown in the figure, the output current is one sinusoidal signal with the same phase as the grid voltage. The voltage of the decoupling capacitor possesses one impulsing part at the double-line frequency, which has a 37V peak-to-peak. This 37V voltage is superposition on an offset value of 110V. It ensures putting the power of the 100 Watts into the grid.

**Fig 16:**
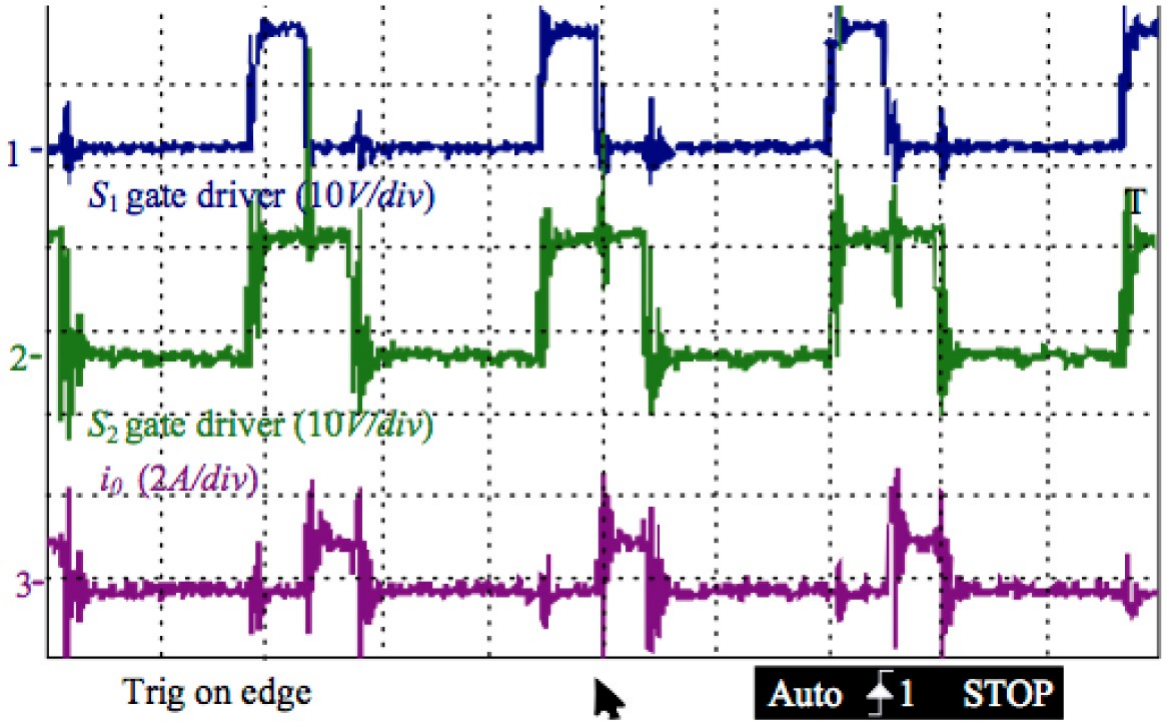
Gate driving voltages of switches S1 and S2, and the output current of the inverter before filtering.

Fig 16 below demonstrates the original output current of the inverter before filtering and the gate driving signals of switches S_1_ and S_2_ for the positive half-side cycle. Switching S_2_ and turning off S_3_ can produce a sinusoidal current in the output after filtering. The duty cycle in S_2_ is unchanged with S_1_ while the voltage of the grid passes by the point of zero. Switch S_2_’s duty cycle reaches its maximum if the outputting voltage locates at the peak point.

**Fig 17:**
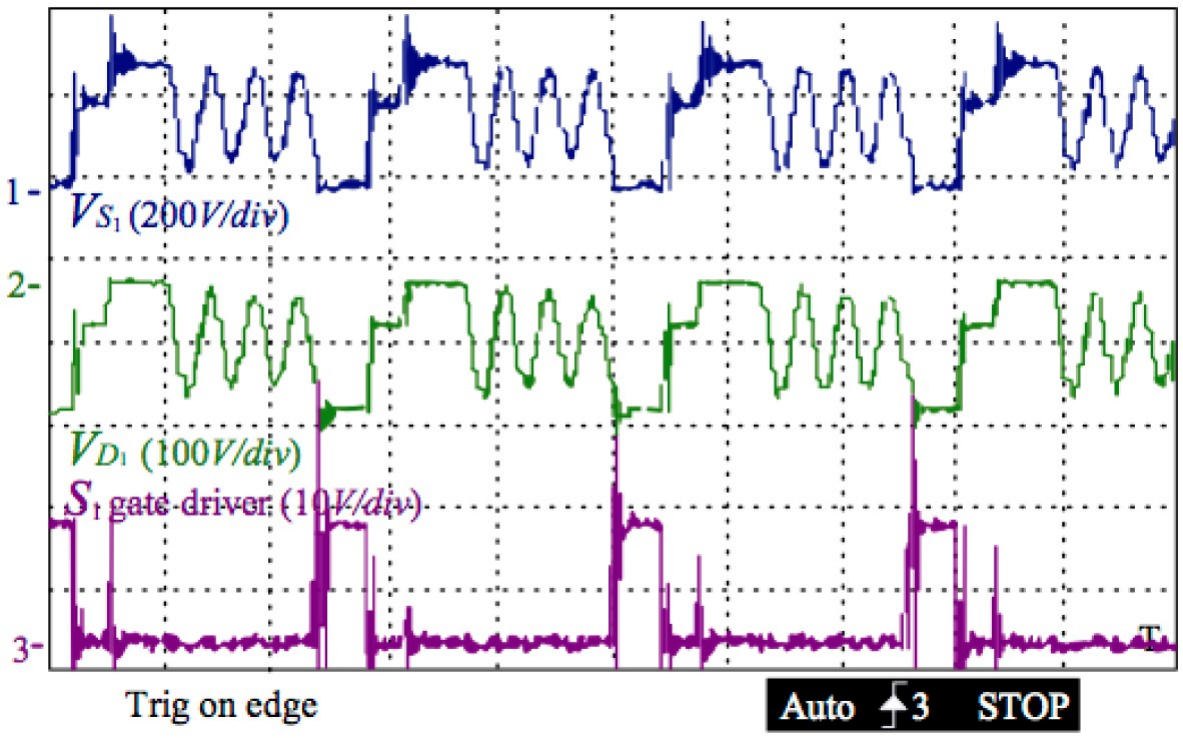
Gate driving voltages of switches S1 and S2, and the output current of the inverter before filtering.

Fig 17 below depicts the voltage of diode D_1_ and the drain-source and gate source of switch S_1_ and S_2_, respectively. The voltage of diode D_1_ meets its maximum of −V_C1_− (V_C1_ + V_PV_)/n_12_ when switch S_1_ is turning on. Turning S_1_ off and S_2_ on can clamp S_1_ voltage to *V*_SS_ and let D1 voltage be −V_C1_ + n_23_*V*_out_, where n_12_ is the turn-ratios of the transformer n_1_ /n_2_, and n_23_ is n_2_ /n_3_.

**Fig 18:**
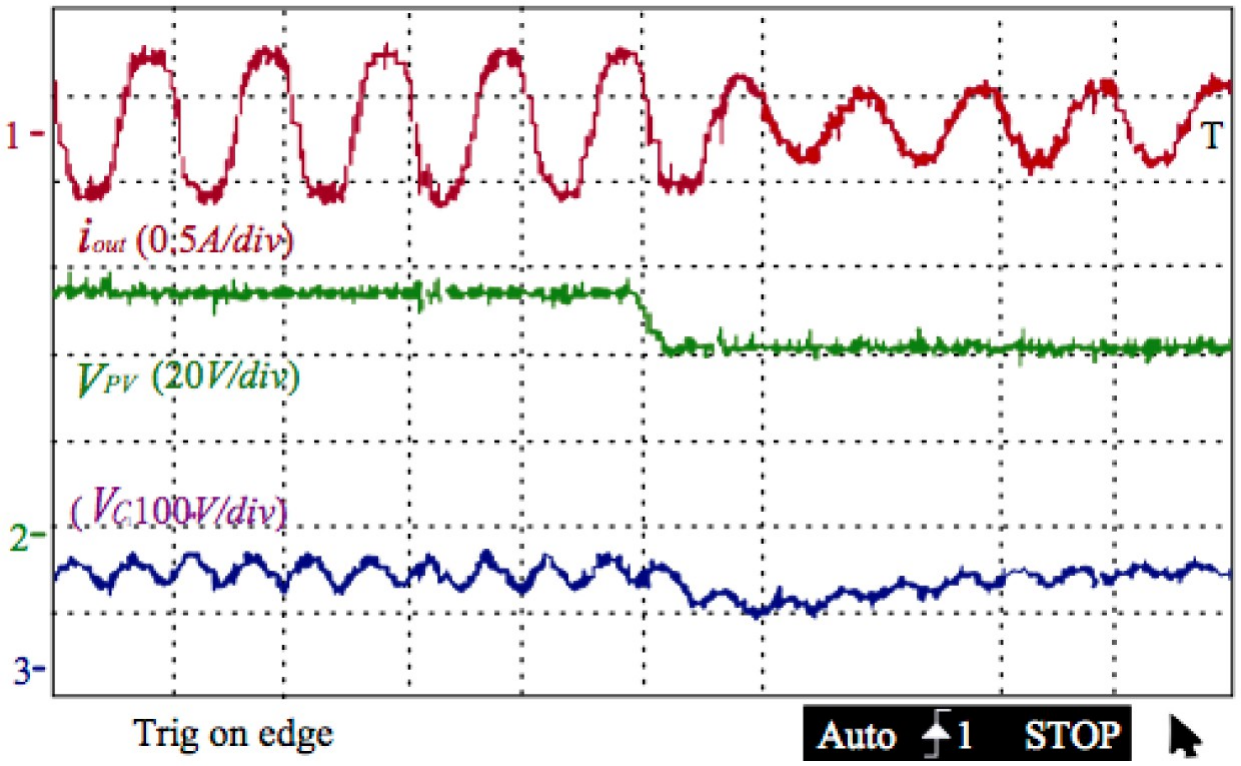
Outputting current, the grid voltage, and the voltage of the decoupling capacitor.

Fig 18 below illustrates the dynamic response of the inverter presented in this article. When the PV voltage goes down to 40V from 55V, the ripple voltage of this decoupling capacitor goes down from 25 to 15V. Meanwhile, the output power decreases to 35W from 62. The controlling method can compensate for the voltage variation of the decoupling capacitor during four periods by improving the output power.

### 5.3. Algorithm efficiency and loss distribution

**Fig 19:**
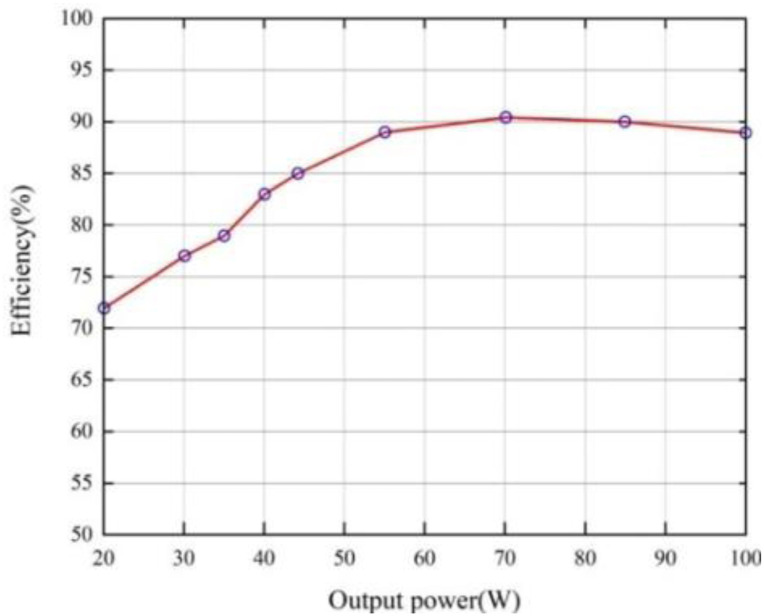
Efficiency of the proposed inverter versus its output power.

Fig 19 below represents the efficiency of the presented inverter with the output power changes. Dividing ***P***_*out*_ by ***P***_*in*_ can compute this efficiency, and multiplying corresponding currents by voltages also can measure the output and input power. This implemented inverter reaches its maximum efficiency point of 91.2% for half of the rated power nearly while realizing the efficiency value of 88.8% at its rated one.

**Fig 20:**
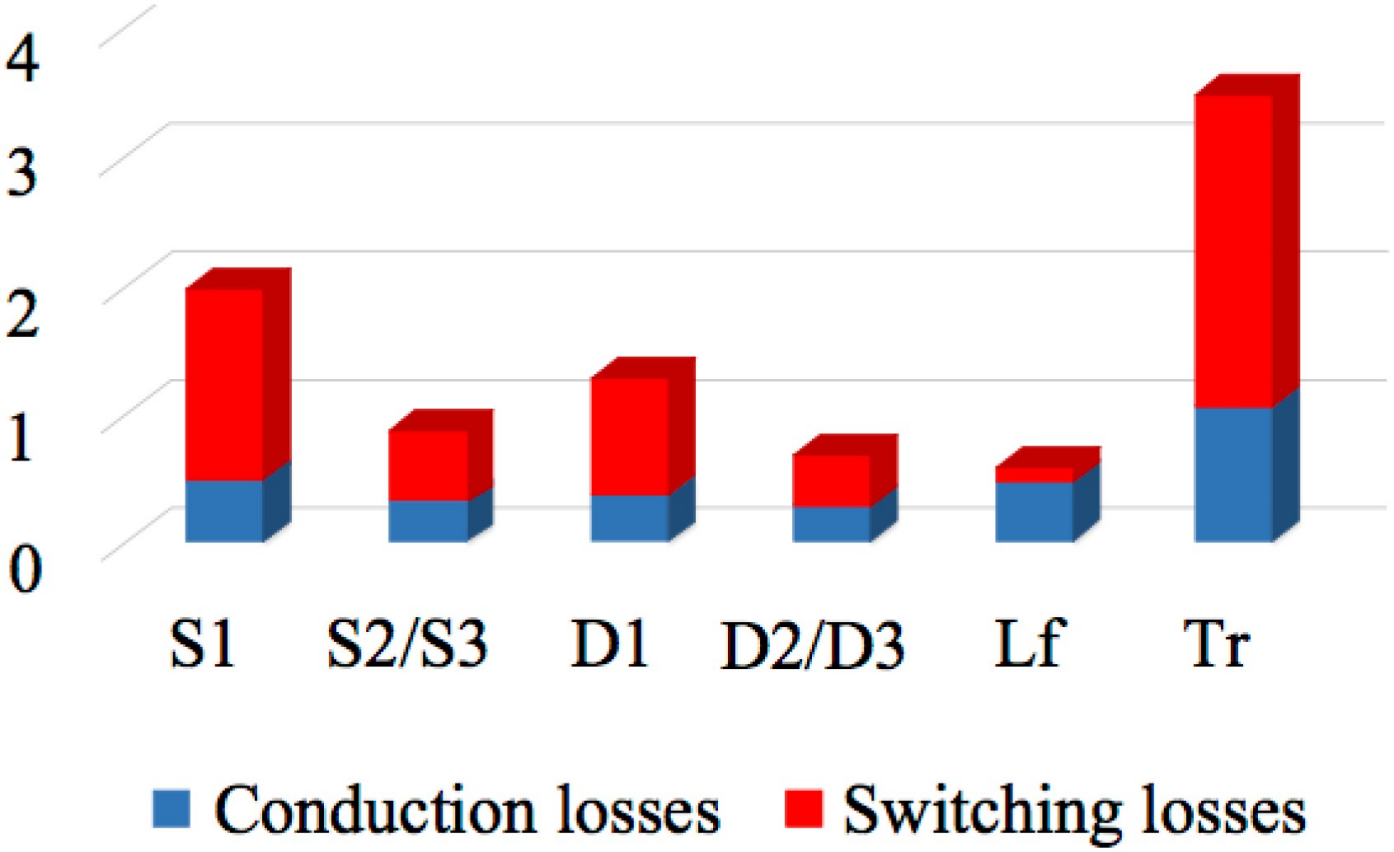
Loss distribution of the main components.

Fig 20 below illustrates the loss distribution of various significant components at the rated power. The loss of diodes, three switches, filter inductance, and flyback transformers is more than 90% of the whole losses. The most important source of the inverter losses is the core loss. Utilizing a larger wire width and a bigger core size could reduce the core loss and optimize the system’s efficiency. Furthermore, some high-flux density(permeability) materials, just as non-crystalline or amorphous wire, can significantly increase the efficiency of the micro inverter while they will cost.

Under the same design conditions, we tested the heat dissipation of the switch inverter system using three different switching devices: F4-23MR12W1M1_B11, F4-100R06KL, and F4-30R06W1E3. Table 4 illustrates that the method proposed in this paper outperforms the traditional H-bridge topology in conduction loss, switching loss, total loss, and heat dissipation performance.

**Table 4:** Comparisons between traditional H-bridge and the proposed topology.

Indexes		Topological types	
Switching devices	Indicators	Traditional H-bridge	Proposed method
**F4-23MR12W1M1_B11**	**Conduction loss/W**	0.4	0.44
	**Switching loss/W**	1.3	0.95
	**Total loss/W**	1.7	1.39
	**Radiator volume ratio**	0.87:1	
**F4-100R06KL4**	**Conduction loss/W**	1.7	2.3
	**Switching loss/W**	3.7	2.7
	**Total loss/W**	5.4	5.0
	**Radiator volume ratio**	0.94:1	
**F4-30R06W1E3**	**Conduction loss/W**	1.6	2.45
	**Switching loss/W**	3.8	2.7
	**Total loss/W**	5.4	5.15
	**Radiator volume ratio**	0.97:1	

NOTE PLEASE: The radiator volume ratio is the ratio between the two topological types, and the proposed method is the value before the colon in the ratio.

As shown in Table 4, this topology reduces the voltage stress on the switching tubes, which is beneficial for selecting high-performance switching tubes with low power levels and further reducing switching losses. According to the Pareto optimality principle, when other parameters remain unchanged, the conclusion that the new topology is superior to the traditional one was validated by comparing the AC power of the inductance and the heat dissipation of the switching devices between the traditional and proposed topologies.

**Fig 21:**
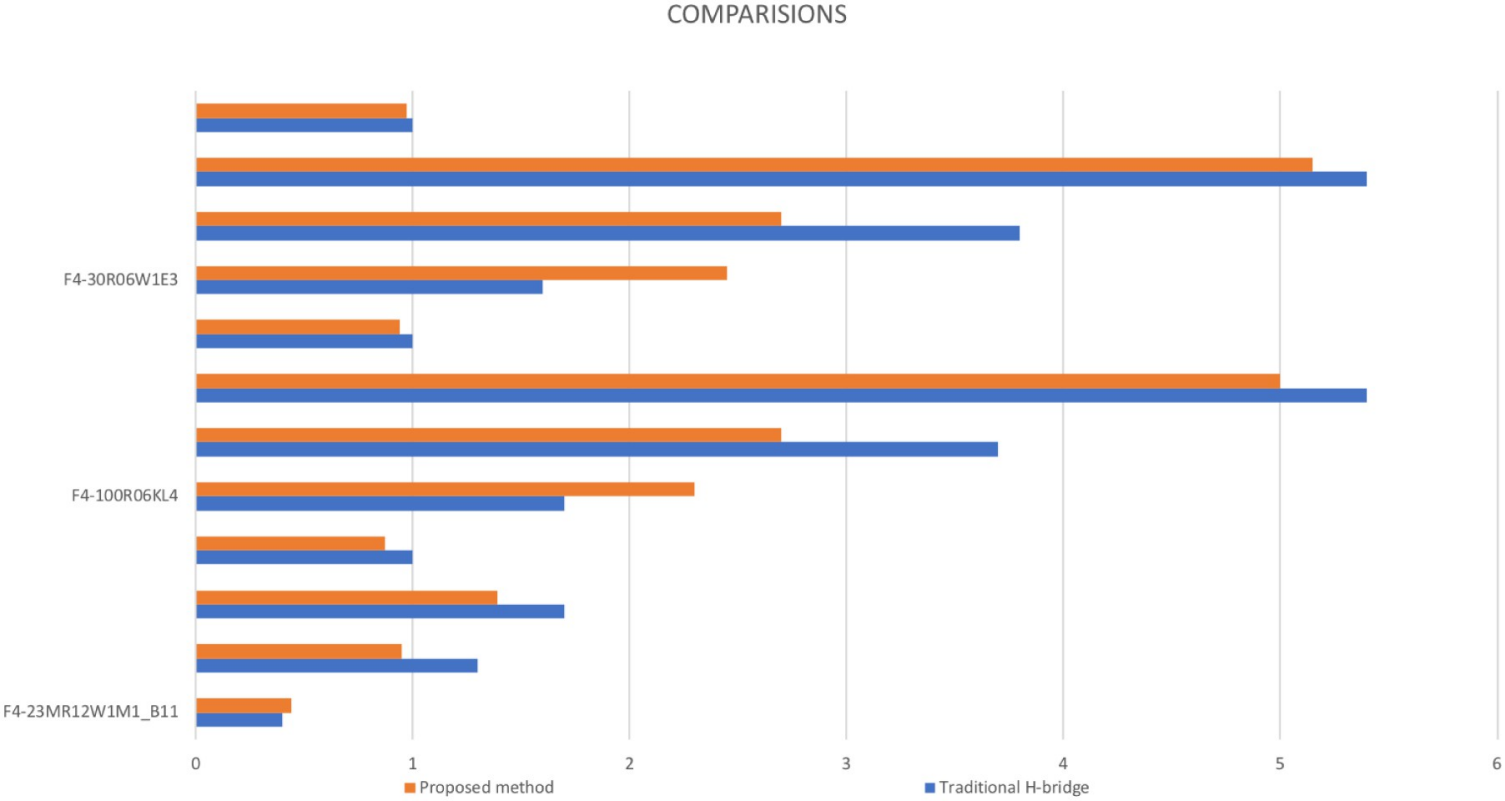
Comparisons profile between traditional H-bridge and the proposed topology.

Besides Table 4, we also created Fig 21 based on the data in Table 4 to enhance our understanding. Three different switching devices mentioned in the table have the same four columns in this figure. The first column represents Conduction loss/*W*, the second column represents Switching loss/*W*, the third column represents Total loss/*W*, and the fourth column represents Radiator volume ratio. This figure illustrates the same conclusion that the method proposed in this article outperforms the traditional H-bridge topology with four switches.

## 6. Conclusion

The grid-connected inverter is an essential component in a PV system, and its performance will be affected by filter inductance, a radiator, an electrolytic capacitor, etc. This article presented a new Boost-type power decoupling inverter based on the principle of power flow optimization to reduce the volume of the filter inductor, decrease the switching loss and improve the power density of the inverter. The proposed power can extract the maximum power point of the PV, deal with ripple power and transfer the low THD-type sinusoidal current into the grid only with three ports and three switches. The simulation and experimental results show that compared with the traditional single-phase inverter topology, under the same experimental conditions, decreasing the number of those active switches can improve the reliability of the micro-inverter through the presented method. The proposed compact, trusty, and economical inverter only have three disjunctors and an easy controlling strategy, so it is a good choice for a PV low-power decentralized application.

Virtually this research can improve the reliability of the micro-inverter by reducing the number of active switches. Although the proposed inverter, a somewhat perfect candidate for the PV low-power decentralized application, is a compact, reliable, and economical method, it has shortcomings, such as plug-and-play operation and extended system efficiency. Therefore, these limitations are our future research.

As for a PV system with an MPPT, like the grid system utilized in our paper, the average voltage of decoupling capacitors increases as the MPPT controller increases its inputting power. The amplitude of the injected current, *I*_*m*_, increases compared to a reference voltage value. Much more grid power will be injected into the system. Therefore, the current *I*_*m*_ is different from before, so the average voltage of decoupling capacitors is close to that reference value. Accordingly, the presented topology is more suitable for low to medium powers (up to moderately high) than for high powers.

## Supporting information

S1 File (DOCX)
