# Supplementary material for: An efficient power decoupling topology circuit based on a novel three-port three-switches flyback series circuit
Source: PLoS One. 2024 Aug 1;19(8):e0305773. doi: 10.1371/journal.pone.0305773 (PMC11293754; doi:10.1371/journal.pone.0305773)
Supplement: S1 File — (DOCX) [file pone.0305773.s001.docx]

## All formulas with the supporting caption

**The All Supporting Figures Caption**

**Eq. (1)** the real-time inputting power in the traditional grid-connected flyback inverter

**Eq. (2)** Instantaneous outputting power traditional grid-connected flyback inverter

**Eq. (3)** the instantaneous power in the buffer traditional grid-connected flyback inverter

**Eq. (4)** the current in the transformer linearly increases from the point of zero in mode1

**Eq. (5)** the voltage of the decoupling capacitor at the end of mode1

**Eq. (6)** the increasing voltage starts from the value of zero and lastly ends with a value in mode 2

**Eq. (7)** the leakage inductance current in the transformer in mode 2

**Eq. (8)** the voltage of switch S_1_ in the transformer in mode 2

**Eq. (9)** a peak value of the outputting current in the transformer at the start of mode 3

**Eq. (10)** a minimum value of the outputting current in the transformer at the start of mode 3

**Eq. (11)** the current of the magnetizing inductance from the starting of mode 4

**Eq. (12)** when mode 4 ends, the voltage of the decoupling capacitor could increase to a voltage*V_c-c_*

**Eq. (13)** the voltage of switch S_1_ in mode 4

**Eq. (14)** the buffer voltage in mode 5

**Eq. (15)** the inductance current in mode 5

**Eq. (16)** the duty cycle of switches (S_2_ or S_3_) equals a sum of d_1_ and d' deduced by equation (9)

**The All Formulas**

$\boldsymbol{P}_{\mathrm{PV}}=V_{\mathrm{PV}}\cdot I_{\mathrm{PV}}$ (1)

$\boldsymbol{P}_{\mathrm{out}}\left( t \right)=V_{m}\cdot I_{m}\sin^{2}\omega t=\boldsymbol{P}_{\mathrm{PV}}\cdot\left( 1-\cos2\omega t \right)$ (2)

${\boldsymbol{P}_{\mathrm{PD}}\left( t \right)\boldsymbol{=P}}_{\mathrm{PV}}\cdot cos2\omega t$ (3)

$i_{Lm}\left( t \right)=\frac{V_{PV}+V_{C_{1}}}{L_{m}+L_{l}}(t-t_{0})$ (4)

$V_{c-b}=\sqrt{V_{c-a}^{2}+\frac{2d_{1}V_{PV}I_{PV}}{C_{1}f}-\frac{L_{m}}{C_{1}}I_{peak11}^{2}}$ (5)

$V_{SS}=V_{C_{1}}+V_{PV}+n_{13}V_{out}$ (6)

$i_{L_{l}}\left( t \right)=i_{peak11}\cos\left( \omega_{0}\left( t-t_{1} \right) \right)+\frac{V_{SS}}{Z_{1}}\sin\left( \omega_{0}\left( t-t_{1} \right) \right)$ (7)

$V_{C_{S1}}=V_{SS}\left[ 1-\cos\left( \omega_{0}\left( t-t_{1} \right) \right) \right]+\left( i_{peak11}Z_{1} \right)\sin\left( \omega_{0}\left( t-t_{1} \right) \right)$ (8)

$i_{peak21}=n_{13}\frac{V_{PV}+V_{C_{1}}}{\left( L_{m}+L_{l} \right)f}d_{1}$ (9)

$i_{\mathrm{peak}22}^{2}=i_{\mathrm{peak}21}^{2}-\frac{2n_{13}^{2}V_{m}I_{m}\sin^{2}\omega t}{fL_{m}}$ (10)

$i_{peak12}=i_{peak22}/n_{13}$ (11)

$V_{c-c}=V_{c-b}+\frac{L_{m}L_{peak12}^{2}}{2n_{12}C_{1}V_{C_{1}}}$ (12)

$V_{\mathrm{dd}}=V_{\mathrm{PV}}+(1+n_{12})V_{C_{1}}$ (13)

$V_{C_{S1}=}V_{pp}+n_{12}V_{C_{1}}cos(\omega_{0}^{'}(t-t_{4}))$ (14)

$i_{L_{l}}\left( t \right)=-(n_{12}V_{C_{1}}/Z_{1}^{'})sin(\omega_{0}^{'}(t-t_{4}))$ (15)

$d^{'}=\frac{L_{m}f}{n_{13}^{2}V_{m}\left| \sin\omega t \right|}\left( i_{\mathrm{peak}21}-i_{\mathrm{peak}22} \right)$ (16)
